# Prospective evaluation of imipenem trough concentrations and clinical outcomes in critically ill patients

**DOI:** 10.1093/jac/dkag242

**Published:** 2026-08-18

**Authors:** Mia Madeleine Lidén, Filippo Boroli, Elodie Von Dach, Pierre Lescuyer, David Tonoli, Angela Huttner, the OPTIMAL TDM Study Group, Abderrahim Karmime, Rosanne Bourgeois-Pfister, Stephan Harbarth, Benedikt Huttner, Roselyne Ing, Dionysios Neofytos, Virginie Prendki, Xavier Roux

**Affiliations:** Paediatric Department, Geneva University Hospitals and Faculty of Medicine, Rue Willy-Donzé 6, Geneva 1205, Switzerland; Intensive Care Unit, Geneva University Hospitals, Rue Gabrielle Perret-Gentil 4, Geneva 1205, Switzerland; Centre for Clinical Research, Clinical Trials Unit, University Hospital and University of Geneva, Rue Gabrielle Perret-Gentil 4, Geneva 1205, Switzerland; Toxicology and Therapeutic Drug Monitoring Laboratory, Geneva University Hospitals, Rue Gabrielle Perret-Gentil 4, Geneva 1205, Switzerland; Toxicology and Therapeutic Drug Monitoring Laboratory, Geneva University Hospitals, Rue Gabrielle Perret-Gentil 4, Geneva 1205, Switzerland; Infectious Disease Division, Geneva University Hospitals and Faculty of Medicine, Rue Gabrielle Perret-Gentil 4, Geneva 1205, Switzerland

## Abstract

**Background:**

Imipenem is a broad-spectrum antibiotic commonly used in critically ill patients, whose pharmacokinetic profile can be highly variable. While imipenem therapeutic drug monitoring (TDM) is being adopted increasingly, efficacy and toxicity plasma thresholds remain incompletely defined. Few studies have prospectively and systematically assessed imipenem concentrations in severely ill patients before clinical failure or toxicity events occur.

**Methods:**

This prospective observational cohort study conducted at the University Hospitals of Geneva consecutively included all adult patients hospitalized for suspected or confirmed severe bacterial infections in the haematology, intermediate care and intensive care units whose clinicians prescribed imipenem. On inclusion, patients underwent imipenem plasma-concentration monitoring, with trough levels measured at steady state. We assessed the frequency of adverse events and clinical success over a 30-day period.

**Results:**

Of 142 patients undergoing imipenem monitoring, 106 had trough levels drawn at steady state. The median trough concentration was 2.4 mg/L (IQR 1.2–4.0). Among all patients, adverse events occurred in 4.9% (7/142), while clinical success was achieved in 88/106 with trough levels (83%). No significant differences in trough levels were observed between patients with and without clinical success or with and without adverse events. Renal clearance had a significant impact on trough levels.

**Conclusion:**

Prospective, universally applied imipenem monitoring reveals lower trough levels than previously described. Nonetheless, this cohort of critically ill and/or immunosuppressed patients still experienced high rates of clinical success, with few adverse events. While low trough concentrations did not predict failure, rare outlying higher concentrations (10–20 mg/L) also did not predict toxicity.

## Introduction

Imipenem is a carbapenem with a wide spectrum of antibacterial activity^1^ and rapid penetration into a broad range of tissues^2^; its high renal clearance, however,^3^ may ultimately lead to lower plasma and tissue concentrations, particularly in patients with severe infections and dynamic pharmacokinetics, such as the critically ill.^4–6^

Imipenem is thought to have a relatively wide therapeutic margin and thus has not routinely been subject to therapeutic drug monitoring (TDM); recent work, however, has demonstrated undetectable and thus potentially subtherapeutic plasma levels in patients with ostensibly normal renal function,^4^ leading more clinicians to use TDM for individualized therapy with increased antibiotic exposure.^7^ It is known that imipenem displays low trough levels in certain populations.^4^ While this may be due in part to imipenem’s relative lack of stability, we are lacking prospective clinical data and knowledge on actual cut offs. Both effectiveness and toxicity are important in this context given the recent findings cited above and the perception that imipenem is the most neurotoxic among carbapenems.^8,9^ The aim of this study was to provide evidence towards the establishment of a therapeutic plasma-concentration range of imipenem ensuring non-toxic clinical effectiveness in real-world patients treated for severe bacterial infections.

## Methods

### Study design and population

This single-centre, prospective cohort study conducted at the University Hospitals of Geneva (HUG), Switzerland, included patients receiving one of the seven beta-lactam antibiotics currently measurable by the hospital’s TDM laboratory (amoxicillin,^10^ cefepime,^11^ ceftazidime, flucloxacillin, imipenem, meropenem and piperacillin). This analysis reports outcomes of patients receiving imipenem. From January 2019 to December 2021, adult patients with suspected or microbiologically confirmed systemic bacterial infection were included from the intensive care, intermediate care including geriatrics and transplant units. Patients with planned transfer out of the hospital, a poor prognosis with life expectancy under 1 week or switch to comfort/palliative care were excluded. There was a 6-month break in recruitment in 2020 due to the SARS-CoV-2 pandemic. The Geneva ethics commission deemed the study to be of a quality-control nature given regular operational use of beta-lactam TDM in this hospital (2018-01830); the requirement for informed consent was waived. The study is registered at www.clinicaltrials.gov (NCT03790631).

### Administration and monitoring

Once included, patients receiving intermittent imipenem infusions prospectively underwent plasma monitoring for intermittent and trough sampling once the antibiotic attained presumed steady state (targeted sampling time: 24 h after start, with a window of 12 to 48 h after start) and were observed thereafter for a period of 30 days starting on day 1 of antibiotic therapy. Clinical outcomes were determined by chart review, telephone contact with patients or with their healthcare providers. Treating physicians were made aware of the study, but did not have direct access to monitoring results, as study-related analyses were not performed in real time but in batch. Clinicians still had access to routine imipenem TDM if requested; any change in imipenem dosing was recorded in the study database.

### Outcomes

The primary outcome was the occurrence of clinical toxicity through to day 30 in all patients and as stratified by trough level. Secondary outcomes were the incidence of clinical success overall and as stratified by trough level, all-cause mortality, incidence of *Clostridioides difficile* infection and trough-level variability among patient groups.

### Definitions

Clinical success was defined as either full resolution of symptoms and signs of infection (vital signs, infectious symptomatology, inflammatory markers such as C-reactive protein and white blood cell count) or clinical improvement in symptoms and signs in the 30-day observation period.

Clinical failure was defined by absence of clinical resolution or improvement, or by mortality deemed possibly, probably or certainly related to the targeted infection.

A trough imipenem level was defined as the plasmatic measurement taken 0 to 30 minutes before the next administered dose of imipenem. An intermediate level was defined as the measurement taken any time between 60 minutes after the last dose of imipenem and >30 minutes before the next dose.

Renal function was calculated using the Cockcroft-Gault equation. Augmented clearance (ARC) was defined by an estimated creatinine clearance (CrCl) equal or superior to 130 mL/min.^12^

### Outcomes assessment

Clinical response and adverse-event causality were assessed by two investigators (M.M.L. and F.B.) independently with a third investigator for disagreement resolution (A.H.). Investigators were blinded to patient’s plasma concentrations throughout all assessments. Adverse events (AE) deemed possibly, probably or certainly related to imipenem were included in the analysis, which used the WHO–Uppsala Monitoring Centre’s causality assessment system.^13^

### Laboratory TDM

Imipenem samples were collected in lithium heparin tubes before being immediately placed on ice. Samples were brought to the laboratory within 1 hour to the laboratory where they were immediately processed, including at night or on weekends. Samples were centrifuged and plasma was stored at −20°C (without addition of a stabilizer) until analysis (<72 h). For long-term storage, plasma samples were kept at −80°C. The analytical method for the measuring of plasmatic imipenem was validated according to Clinical and Laboratory Standards Institute (CLSI) guidelines and is detailed in the Supplementary Data (Tables S1 and S2, available as Supplementary data at *JAC* Online). As minimal inhibitory concentrations (MIC) were not performed routinely, we used EUCAST breakpoints for Enterobacterales and *Pseudomonas* spp.

### Statistical analysis

#### Sample size

The target sample size was a minimum of 100 patients with at least one imipenem trough concentration correctly drawn and processed. This was a convenience sample determined chiefly by budgetary and logistical considerations.

#### Study populations

All patients had only one trough level drawn; some patients had one trough level and one intermediate level drawn. While all patients with any imipenem plasma-level monitoring, whether trough or intermediate levels, were included in the safety population for assessment of AE, only patients receiving intermittent dosing with trough-level sampling were included in the ‘effectiveness’ population for assessment of clinical success. This report focusses primarily on the latter population given the clinical practice of trough-level monitoring for beta-lactam antibiotics,^7^ in contrast to the heterogeneity and uncertain clinical interpretation of intermediate levels.

#### Analyses

We describe continuous variables with medians and interquartile ranges (IQR), and categorical variables with count and percentages. Values reported by the laboratory to be <0.5 mg/L were considered to be half (i.e. 0.25 mg/L) the detection limit for statistical analysis in accordance with guidelines.^14,15^ We assumed the association of outcome between groups with *χ*^2^, Fisher’s exact, Mann–Whitney and Kruskal–Wallis tests, as appropriate. Associations with a *P* value ≤0.05 were considered to be significant. Univariable and multivariable logistic regressions were used to explore associations between baseline characteristic variables and trough levels. The results of the latter analyses were derived as odds ratios with 95% confidence intervals. Data were collected and stored in REDCap,^16,17^ and analyses were performed in R, using RStudio.

## Results

### Baseline characteristics

In total, 142 patients treated with imipenem and undergoing any plasma drug monitoring were included (safety population). Of these, 106 received standard intermittent dosing and had one correctly drawn trough level once in steady state (‘effectiveness’ population; Figure S1).

Patients with trough levels were predominantly male (69/106, 65.1%) and Caucasian (103/106, 97.2%) with a median age of 63 years (IQR 53–73) and a median qSOFA score at the start of therapy of 1 (IQR 0–2). Only a few were immunocompetent (58/106, 54.7%); source of infection was undetermined in 38/105 (36%), pulmonary in 29 (27.4%) and abdominal in 13/105 (12.3%). Median renal clearance at onset of infection was 80.1 mL/min (IQR 46.5–121.4). Other baseline characteristics are described in Table 1. All patients were followed for the 30-day period (or until death if before day 30).

**Table 1.** Baseline characteristics of the 106 patients with an available imipenem plasmatic trough level stratified by development of an adverse event over the study period. Percentages in the ‘all patients column’ illustrate the distribution of all patients between groups. The percentage in the ‘patients with AE’ and ‘Patients without AE’ columns illustrate the different groups between patients presenting with an AE and those not presenting with an AE

	All patients *n* = 106	Patients with AE *n* = 4 (3.8%)	Patients without AE *n* = 102 (96.2%)	*P*
Gender (%)				0.5
Female	37 (34.9)	2 (5.4)	35 (94.6)	
Male	69 (65.1)	2 (2.9)	67 (97.1)	
Age, years median (IQR)	64 (53–73)	78 (66.5–83.5)	64 (53–72)	0.2
Ethnicity (%)				1.0
Caucasian	103 (97.2)	4 (3.9)	99 (96.1)	
African	3 (2.8)	0 (0)	3 (100)	
BMI median (IQR)	24.2 (20.3–26.9)	24.6 (21.9–25.9)	24.2 (20.3–27.2)	0.8
qSOFA (%)				0.5
0	36 (34.0)	2 (5.6)	34 (94.4)	
1	20 (18.9)	0 (0)	20 (100)	
2	39 (36.8)	1 (2.6)	38 (97.4)	
3	11 (10.4)	1 (9.1)	10 (90.9)	
Hospital unit (%)				0.1
Haematology	38 (35.8)	3 (7.9)	35 (92.1)	
Intensive care unit	39 (36.8)	0 (0)	39 (100)	
Intermediate care unit	29 (27.4)	1 (3.4)	28 (96.6)	
Immunosuppression (%)				1.2
Yes	48 (45.3)	2 (4.2)	46 (95.8)	
No	58 (54.7)	2 (3.4)	56 (96.6)	
Indication for imipenem (%)				0.3
Unknown	38 (35.8)	1 (2.6)	37 (97.4)	
Pneumonia	29 (27.4)	1 (3.4)	28 (96.6)	
Abdominal infection	13 (12.3)	0 (0)	13 (100)	
Urinary tract infection	8 (7.5)	0 (0)	8 (100)	
Catheter-associated infection	6 (5.7)	1 (16.7)	5 (83.3)	
Wound infection	3 (2.8)	0 (0)	3 (100)	
Neutropenic fever	2 (1.9)	0 (0)	2 (100)	
Other^a^	6 (5.7)	1 (16.7)	5 (83.3)	
Missing	1 (0.9)	0 (0)	1 (100)	
Creatinine clearance mL/min (Cockcroft–Gault) median (IQR)	80.2 (46.5–121.4)	75.5 (60.4–122.0)	80.2 (46.9–120.8)	0.8
<15	2 (1.9)	0 (0)	2 (100)	1
15–29	7 (6.6)	0 (0)	7 (100)	
30–44	29 (27.4)	1 (3.4)	28 (96.6)	
45–59	24 (22.6)	1 (4.2)	23 (95.8)	
60–89	19 (17.9)	0 (0)	19 (100)	
≥90	22 (20.8)	1 (4.5)	21 (95.5)	
Missing (%)	3 (2.8)	1 (33.3)	2 (66.7)	
Concomitant antibiotic during the study period (%)				0.6
Yes	84 (79.2)	4 (4.8)	80 (95.2)	
No	22 (20.8)	0 (0)	22 (100)	
Concomitant antibiotic at time of trough-level sampling (%)				1
Yes	34 (32.1)	1 (2.9)	33 (97.1)	
No	72 (67.9)	3 (4.2)	69 (95.8)	

^a^Other: Fournier’s gangrene (one), hip infection (one), infectious endocardis (one), prophylaxis (one), COVID (one), skin infection (one)

### Imipenem dosing and TDM

The median starting dose was 2.82 g/day (IQR 1.4–4.4 g/day) with most patients receiving 2 g (81/106, 76.4%). Doses were changed in 16 patients, with reductions in nine patients, all of whom had CrCl <60 mL/min. Median duration of therapy was 6 days (IQR 3–10); imipenem was often used initially as empirical therapy and replaced by more targeted therapy as culture results became available.

Median time between start of antibiotic therapy and TDM was 26.5 hours (IQR 17.5–41.0) (Figure S2). Imipenem trough levels were generally low [median concentration was 2.4 mg/L (IQR 1.2–4.0)]. Five (4.7%) patients had undetectable trough levels. All five patients received 500 mg/6 h of imipenem, four (80%) had ARC and four (80%) were hospitalized on the haematology ward. For two of these patients (40%), imipenem was given for an unknown infection, for one (20%) neutropenic fever, for one (20%) an abdominal infection and the last one (20%) for a urinary tract infection.

There was little inter-patient variability in trough levels. No significant difference was observed between sexes (*P* = 0.5), elderly and non-elderly (*P* = 0.7), immunosuppressed and not (*P* = 0.1) or between doses (*P* = 0.6) (Table 2, Figure S3).

**Table 2.** Median plasmatic imipenem trough level (mg/L) overall and stratified by presence of AE in the observation period, clinical success in the observation period, clinical outcome groups, sex, age group, immunosuppression, hospital unit, imipenem dose and creatinine clearance

	Median (mg/L)	P25, P75	*P* value
All (*n* = 106)	2.4	1.2, 4.0	
AE			0.9
Yes (*n* = 4)	2.8	1.4, 4.4	
No (*n* = 102)	2.4	1.2, 3.9	
Clinical success			1.0
Yes (*n* = 84)	2.4	1.2, 4.1	
No (*n* = 22)	2.4	1.3, 3.9	
Clinical outcome groups			0.6
Clinical cure (*n* = 70)	2.44	1.3, 4.1	
Clinical improvement (*n* = 14)	1.5	0.8, 3.1	
Clinical failure (*n* = 22)	2.4	1.3, 3.88	
Sex			0.5
Male (*n* = 69)	2.5	1.2, 4.2	
Female (*n* = 37)	1.7	1.2, 3.7	
Age (years)			0.7
<85 (*n* = 101)	2.4	1.2, 4.0	
≥85 (*n* = 5)	2.3	1.7, 2.4	
Immunosuppression			0.1
Yes (*n* = 48)	1.8	1.0, 3.8	
No (*n* = 58)	2.5	1.4, 4.2	
Hospital unit			0.004
Intensive care (*n* = 39)	2.8	1.3, 4.8	
Haematology ward (*n* = 38)	1.3	0.9, 2.9	
Intermediate care (*n* = 29)	3.0	1.9, 4.0	
Imipenem dose			0.6
500 mg/6 h (*n* = 81)	2.4	1.2, 4.0	
500 mg/8 h (*n* = 5)	4.2	1.4, 5.1	
500 mg/12 h (*n* = 4)	1.3	0.9, 1.9	
750 mg/6 h (*n* = 5)	3.3	1.5, 3.5	
750 mg/8 h (*n* = 4)	2.8	2.3, 9	
750 mg/12 h (*n* = 2)	4.9	4.8, 5.0	
1 g/24 h (*n* = 1)	1.9	NA	
1 g/8 h (*n* = 1)	1.4	NA	
1 g/6 h (*n* = 1)	3.7	NA	
250 mg/6 h (*n* = 1)	2	1.6, 2.4	
Daily dose			0.9
≥ 2 g (*n* = 92)	2.4	1.2, 3.8	
< 2 g (*n* = 14)	2.2	1.3, 4.6	
Creatinine Clearance mL/min (Cockcroft-Gault) median (IQR) (Missing = 3)			0.0001
<15 (*n* = 2)	2.7	2.5, 2.9	
15–29 (*n* = 7)	2.8	1.4, 4.9	
30–44 (*n* = 29)	3.1	2.4, 5.1	
45–59 (*n* = 24)	2.4	1.4, 4.1	
60–89 (*n* = 19)	1.4	1.0, 2.6	
≥90 (*n* = 22)	1.2	0.7, 2.6	
Augmented renal clearance Creatinine clearance >130 mL/min/(missing = 3)			0.01
Yes (*n* = 22)	1.2	0.6, 2.6	
No (*n* = 81)	2.6	1.4, 4.2	
Concomitant antibiotic during the study period			0.8
Yes (*n* = 84)	2.4	1.6, 3.18	
No (*n* = 22)	2.5	1.1, 4.2	
Concomitant antibiotic at time of trough-level sampling			0.5
Yes (*n* = 34)	2.8	1.2, 4.7	
No (*n* = 72)	2.4	1.2, 3.5	

There was, however, significant variability among patients in different units of the hospital. Patients in the ICU had a median trough concentration of 2.8 mg/L (IQR 1.3–4.8), those in the haematology ward 1.3 mg/L (IQR 0.9–2.9) and those in step-down units 3.0 mg/L (IQR 1.9–4.0). Renal clearance in each ward had similar variability with median of 111 mL/min (IQR 83.1–143), 71.9 mL/min (IQR 42–123) and 46.7 mL/min (IQR 37.2–61.2) in the haematology ward, the ICU and intermediate care units, respectively.

**Figure 1.**
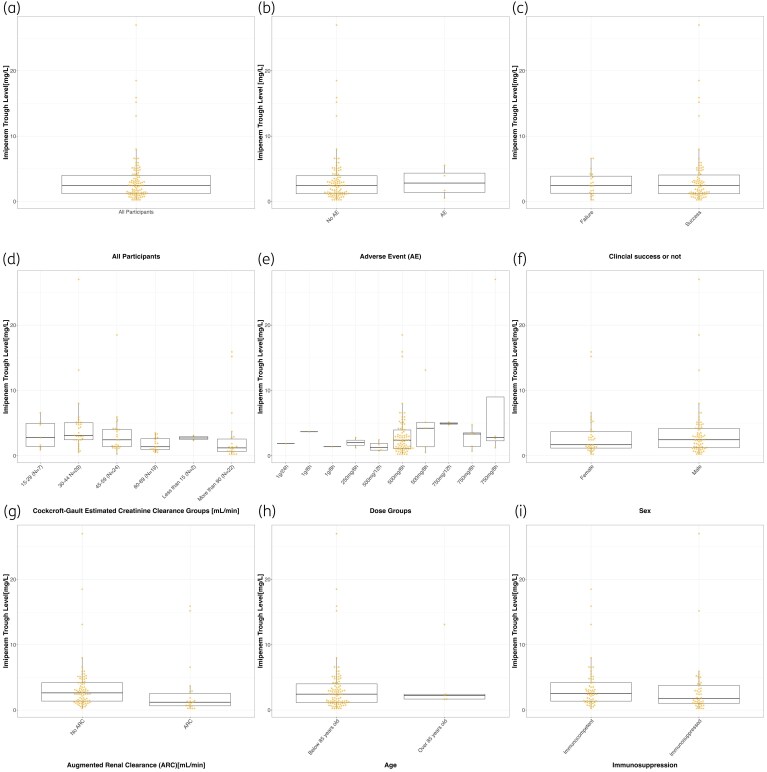
Boxplots of median imipenem trough level by outcome, demographic and clinical group: (a) all participants, (b) by adverse-event occurrence, (c) by clinical outcome, (d) by Cockcroft-Gault estimated creatinine clearance group, (e) by dose group, (f) by sex, (g) by presence of ARC, (h) by age group and (i) by immunosuppression status.

Unsurprisingly, there was an inverse relationship between estimated CrCl and trough concentrations (Figure 1, Table 2, Figure S3). Patients with ARC had significantly lower trough levels [1.2 mg/L (IQR 0.6–2.6) versus 2.6 mg/L (IQR 1.4–4.2), *P* = 0.01] (Table 2). This was confirmed by logistic regression with 4.3-fold odds of being the lowest trough quartile for patients with ARC when compared with those without (95% CI 1.6–13.1, *P* = 0.01).

### Outcomes

#### Toxicity

Overall, seven AE were documented in 142 (4.9%) patients with any TDM performed. In those with trough concentrations, there were four AE over the observation period (Table 3). All were considered only possibly related to imipenem therapy.

**Table 3.** Description of the type, severity and causality of the AE occurring during the study period for (a) the participants for whom a plasmatic imipenem trough level was available (106 participants) and (b) the whole study population (142 participants)

	AE type	Severity	Causality		AE type	Severity	Causality
(a) Trough-level patients (*n* = 4)	− Change in mental status (*n* = 1)	Moderate	Possible	(b) Whole population (*n* = 7)	− Change in mental status (*n* = 1)	Moderate (*n* = 1)	Possible
	Diarrhoea (*n* = 1)	Moderate	Possible		Diarrhoea (*n* = 2)	Moderate (*n* = 1) Mild (*n* = 1)	Possible
	Nausea and/or vomiting (*n* = 1)	Mild	Possible		Nausea and/or vomiting (*n* = 1)	Moderate (*n* = 1)	Possible
	Rash (*n* = 1)	Mild	Possible		Rash (*n* = 3)	Mild (*n* = 2) Life-threatening (*n* = 1)	Possible

There were no significant differences in AE occurrence by demographic or clinical group. Those with trough levels in the highest quartile did not experience more toxicity (1/27, 3.7% versus 3/79, 3.8%, *P* = 1.0). Indeed, univariable logistic regression showed odds ratios of 1.0 for AE occurrence with every 1.0 mg/L increase in trough level (95% CI 0.6, 1.2) illustrating the lack of association.

#### Clinical success

Eighty-four of the 106 patients with trough levels (79.3%) had clinical success, among them 70 (83%) full resolution and 14 (17%) clinical improvement. Median time to success was 11.7 days (IQR 5.6–18.2). Clinical success and time to resolution did not differ significantly by imipenem trough-level groups (Table 4). Furthermore, there was not a significant difference in days to resolution when comparing patients with undetectable trough levels (Table 4).

**Table 4.** Success and median days to success stratified by trough-level groups

	Success *n* (%)	*P* value	Median number of days to success (IQR)	*P* value
Detectable though level (*n* = 101)	81 (80.1)	0.28	11.7 (11.9–13.5)	0.7
Undetectable trough level (*n* = 5)	3 (60)		12.7 (11.9–13.5)	
Q3 of trough level (*n* = 27)	22 (81.5)	1	14.5 (5.3–19.7)	0.23
Under Q3 trough level (*n* = 79)	62 (78.5)		10 (6.1–17.2)	

We did not observe a difference in trough levels between patients with clinical success and failure [2.4 mg/L (IQR 1.2–4.1) versus 2.4 mg/L (IQR 1.3–3.9), *P* = 1.0] (Table 2, Figure S3). There were also no significant differences in trough levels between patients who already had an AE (2.8, 1.4, 4.4) and those who did not [2.4 mg/L (IQR: 1.2–3.9), *P* = 0.9] (Table 2, Figure S3).

#### Clostridioides difficile *infection*

Two participants had a confirmed or suspected *C. difficile* infection during the observation period.

#### Death

There were 14 deaths over the observation period. For eight (57.1%) of the patients, the cause of death was the infection being treated by imipenem; for two (14.3%), the cause of death was considered unrelated to the infection; one (7.1%) died of a different infection arising in the observation period; three (21.4%) did not have a cause of death specified.

### Microbiological results

In those with trough levels, 52/106 (49.1%) had 166 positive microbiological samples (Table S3). Forty-nine (29.5%) of these revealed *Escherichia coli*; 4/49 (8.2%) strains were resistant to imipenem. There were 19 positive *Pseudomonas* spp. samples, three of which (15.8%) were resistant strains, including to imipenem.

Ninety-seven (58.4%) samples were from blood, 12/166 (7.2%) from sputum, 12/166 (7.2%) from bronchoalveolar lavage, 8/166 (4.8%) from wound swabs and 8/166 (4.8%) from urine. The remaining 27/166 (16.3%) samples derived from other swab types or drains.

## Discussion

This prospective cohort of 142 critically ill and/or immunosuppressed patients undergoing uniformly applied imipenem plasma monitoring reveals low clinical toxicity and relatively high clinical success rates (5% and 80%, respectively). Indeed, toxicity was rare in this cohort, with only seven events among 142 patients; all seven were deemed only possibly (not probably or certainly) related to imipenem. The events were reversible and, importantly, rarely neurological (*n* = 1).

In the 106 patients with trough-level monitoring, levels were overall lower than those previously described in the literature.^5,18^ Indeed, our recent retrospective study—where the decision to perform TDM was left to the clinician—found a median imipenem trough level of 3.6 mg/L (IQR 1.9–6.6), higher than that in the present study (2.4 mg/L, IQR 1.2–4.0) despite similar imipenem doses.^5^ Thus when applied prospectively and uniformly across all patients (thereby avoiding confounding by indication), plasma monitoring of imipenem reveals a different picture.

Clinically, we were unable to define strict therapeutic concentration thresholds for toxicity and efficacy given the low adverse-event rate and relatively high clinical success rate. Even the rare outlying higher trough concentrations (10–20 mg/L) were not associated with clinical toxicity. And although trough levels varied by renal function, with patients with ARC experiencing significantly lower concentrations as previously described in the literature,^4,11^ no impact was observed on clinical outcome. Indeed, patients with undetectable trough levels did not show more clinical failure. This may be partially explained by (i) the fact that some patients did not have a confirmed bacterial infection, making their outcomes unlikely to be determined by antibiotic therapy in any case; (ii) the relative paucity of pathogens with high MIC in our centre and (iii) the relatively small number of failure events in this cohort, which reduces statistical power to confirm correlations.

Interestingly, even patients with renal insufficiency—whose dosing was appropriately adjusted—experienced low trough levels, raising the question of potential over-adjustment in this population. Given that observation and previous population pharmacokinetic modelling of amoxicillin concentrations in similar patients suggesting that, indeed, on-label renal dosing may be overshooting the mark for that beta-lactam,^19^ we recently applied previous population pharmacokinetic modelling to this study’s cohort of patients. In an international collaboration pooling the current data with those of Czech and Vietnamese patients, we showed only low probabilities of target attainment in those with on-label dose reductions due to renal insufficiency.^20^

Although this real-world study’s limited statistical power to infer strict upper and lower therapeutic plasma thresholds is its main limitation, there are others. Because of budget and other logistic constraints (including frequent transfers between units due to patients’ dynamic clinical courses), serial plasma-concentration levels could not be obtained universally. Reflecting real-world clinical practice, plasma samples could be collected by different nurses per usual standards, which could have introduced small differences in timing of collections and thereby a potential for inter-patient variability. Furthermore, it included all patients, even those without microbiologic confirmation of infection; although some would argue that these two points are a strength, as excluding such patients or having external team for collection of samples would not allow the study to mirror our clinical reality, thereby decreasing its external validity.

### Conclusions

While strict thresholds for toxicity and efficacy could not be determined, this prospective cohort study applying universal trough-level assessment in critically ill and/or immunosuppressed patients found lower imipenem concentrations than previously described (median 2.4 mg/L). These low levels did not, however, predict clinical failure, nor did the rare, outlying higher concentrations (10–20 mg/L) predict clinical toxicity.

## Supplementary Material

dkag242_Supplementary_Data

## Supplementary data

Figures S1 to S3 and Tables S1 to S3 are available as Supplementary data at *JAC* Online.
