# Supplementary material for: Prospective evaluation of imipenem trough concentrations and clinical outcomes in critically ill patients
Source: J Antimicrob Chemother. 2026 Aug 18;81(9):dkag242. doi: 10.1093/jac/dkag242 (PMC13482977; doi:10.1093/jac/dkag242)
Supplement: Supplementary file 1 [file dkag242_supplementary_data.docx]

Prospective Evaluation of Imipenem Trough Concentrations and Clinical Outcomes in Critically Ill Patients

Mia Madeleine LIDÉN^1,*^, Filippo BOROLI, Elodie VON DACH^3^, Pierre LESCUYER^4^, David TONOLI^4^, Angela HUTTNER^5^ for the OPTIMAL TDM Study Group‡

Supplement

Concentrations of imipenem were measured by ultra-high performance liquid chromatography-tandem mass spectrometry (UHPLC-MS/MS). The analytical method is an internal assay measuring total drug concentration developed and validated in the laboratory on the following criteria according to Clinical and Laboratory Standards Institute (CLSI) guidelines: linearity, precision and accuracy on inter- and intra-batch series, lower limit of quantification (LLOQ), dilution integrity, carry-over, matrix effects, interferences, and stability studies. The analytical measurement interval is from 0.5 to 100 mg/L. Samples collected overnight or on weekends were centrifuged and plasma was stored at -20°C until time of analysis. Results of TDM were available to clinicians.

Data were obtained on a Sciex QTRAP4500MD mass spectrometer hyphenated to an Agilent 1260 HPLC. During method validation, intra-day precision and accuracy were evaluated at 3 levels (n=20) with results as follows:

Table S1

| Nominal concentration (mg/L) | Mean measured concentration (mg/L) (n=20) | Accuracy (%) | CV (%) |
| --- | --- | --- | --- |
| 0.500 | 0.521 | 104 | 3.8 |
| 25.0 | 25.9 | 103 | 2.6 |
| 100 | 105 | 105 | 2.7 |

Inter-day precision and accuracy were evaluated at 6 levels (n=40) with results as follows:

Table S2

| Nominal concentration (mg/L) | Mean measured concentration (mg/L), n=40 | Accuracy (%) | CV (%) |
| --- | --- | --- | --- |
| 0.500 | 0.463 | 92.6 | 10 |
| 1.00 | 0.957 | 95.7 | 6.4 |
| 2.50 | 2.396 | 95.9 | 5.5 |
| 5.00 | 4.891 | 97.8 | 5.9 |
| 25.0 | 23.79 | 95.2 | 7.2 |
| 100 | 94.2 | 94.2 | 6.1 |

In the context of routine work, accuracy is also ascertained by participation in a proficiency testing program (ASQUALAB).

Table S3: distribution of the infecting pathogens sampled in the 106 participants for whom a trough level was available

| Pathogen identified | Positive specimens (%) (N=166) | Number of strains resistant to imipenem (%) |
| --- | --- | --- |
| *Escherichia coli* | 49 (29.5) | 4 (8.2) |
| *Pseudomonas* spp. | 19 (11.4) | 3 (15.8) |
| *Enterococcus* spp. | 17 (10.2) | 0 (0) |
| *Klebsiella* spp. | 14 (8.4) | 1 (7.1) |
| *Staphylococcus aureus* | 5 (3.0) | 0 (0) |
| *Proteus* spp. | 5 (3.0) | 0 (0) |
| *Enterobacter* spp. | 3 (1.8) | 0 (0) |
| MRSA | 3 (1.8) | 0 (0) |
| Other* | 51 (30.7) | 3 (5.9) |

* Other: *Acinetobacter baumannii complex, Bacteroides faecis, Bacteroides thetaiotaomicron, Candida albicans, Candida glabrata, Candida tropicalis, Clostridium tertium, Corynebacterium spp., Eggerthella lenta, Fusobacterium spp., Fusobacterium nucleatum, Granulicatella adiacens, Haemophilus parainfluenzae, Hafnia alvei, Klebsiella oxytoca/Raoultella spp., Pantoea septica, Propionibacterium acnes, Propionibacterium spp., Pseudomonas putida, Pseudomonas putida complex, Serratia marcescens, Serratia marcescens complex, Staphylococcus epidermidis, Staphylococcus lugdunensis, Staphylococcus oralis, Staphylococcus warneri, Streptococcus mitis, Streptococcus parasanguinis, Alcaligenes faecalis, Bacteroides ovatus, Bacteroides vulgatus, Stenotrophomonas maltophilia*

Figure S1 : study flow chart

142 Imipenem receving patients and undergoing plasma concentration monitoring

36 patients without trough levels excluded from the effectiveness analyses

106 patients with at least one imipenem trough level at steady state completed follow-up and were included in anaysis

Figure S2: scatterplot of time from start of imipenem treament to trough level in hours by trough level [mg/L]. The different color represent the imipenem dose groups.

Figure S3: scatterplot of Cockcroft-Gault estimated creatinine clearance [mL/min] by imipenem trough level [mg/L]. The different colors represent the imipenem dose groups.
